# Are serum thrombomodulin and interleukin-8 levels associated with disease severity and mortality in critically ill children with respiratory failure?

**DOI:** 10.55730/1300-0144.5896

**Published:** 2024-05-22

**Authors:** Dilan AKGÜN ÜNLÜ, Hazal Ceren TUĞRUL, Selen Ceren ÇAKMAK, Gürkan ATAY, Seher ERDOĞAN

**Affiliations:** 1 Department of Pediatrics, Health Science University, Ümraniye Research and Training Hospital, İstanbul, Turkiye; 2 Department of Pediatric Critical Care, Health Science University, Ümraniye Research and Training Hospital, İstanbul, Turkiye

**Keywords:** Acute respiratory distress syndrome, interleukin-8, thrombomodulin

## Abstract

**Background/aim:**

Thrombomodulin (TM) is found on endothelial cell surfaces and increases in response to endothelial injury of different organs. Interleukin (IL)-8 regulates pulmonary inflammation. TM and IL-8 are candidate biological markers of acute respiratory distress syndrome (ARDS). The aim of the present study was to compare TM and IL-8 levels in pediatric patients with and without ARDS who received respiratory support and to determine their relationships with prognosis.

**Materials and methods:**

This was a prospective observational study of 55 patients who received respiratory support in the pediatric intensive care unit. Eighteen patients without active infection were defined as the control group. Two blood samples were taken for serum IL-8 and TM levels on the first and third days of respiratory support.

**Results:**

The patient group had significantly higher IL-8 and TM levels than the control group [median IL-8: 102.7 (IQR: 180.42–189.47) vs. 45.4 (55.14–70.49) ng/L, p = 0.011; median TM: 6.9 (6.83–9.18) vs. 3.4 (3.62–5.05) ng/mL, p = 0.021]. Patients with ARDS had significantly higher marker levels on the first and third days than those who did not have ARDS. The TM and IL-8 levels of deceased patients were significantly higher than those of the survivors on the first day. In mortality prediction, the cut-off point for IL-8 was found to be >154.7 ng/L, which had sensitivity of 76.9% and specificity of 73.8%. The cut-off point for TM was >8.4 ng/mL, which had sensitivity of 76.9% and specificity of 66.7%.

**Conclusion:**

In our study, higher marker levels correlated with impaired oxygenation and higher mortality. Higher TM and IL-8 levels in ARDS might reflect the degree of vascular injury and inflammation.

## 1. Introduction

Acute respiratory distress syndrome (ARDS) involves acute lung injury affecting both lungs, which results from the disruption of the alveolocapillary membrane and is characterized by diffuse infiltration of the lungs [1]. Lung injury and inflammation become widespread with cytokine activation and the release of proinflammatory mediators [2]. It has been shown that higher interleukin (IL)-8 levels measured at the beginning of ARDS correlate with a worse prognosis and increased mortality [3,4]. Thrombomodulin (TM) is found on endothelial cell surfaces and increases in response to endothelial injury of different organs. However, it is mostly expressed in the lungs. Increased plasma TM levels reflect inflammation, endothelial injury, and a tendency of thrombosis [5]. TM is a candidate biological marker for respiratory failure and ARDS.

Our primary aim in conducting the present study was to compare TM and IL-8 levels in pediatric patients with respiratory failure with or without ARDS who received respiratory support with mechanical ventilation and to determine their relationships with prognosis. Our secondary aim was to evaluate the relationships between levels of TM and IL-8 and the oxygenation index (OI), extrapulmonary organ failure, number of days on mechanical ventilator, and number of days spent in the pediatric intensive care unit (PICU). We also compared IL-8 and TM levels between patient groups with and without ARDS to determine whether they are risk factors for developing ARDS.

## 2. Materials and methods

### 2.1. Study design

A total of 55 patients, including 34 patients receiving invasive mechanical ventilator (IMV) support and 21 patients receiving noninvasive mechanical ventilator (NIMV) support due to respiratory failure lasting longer than 72 h in the PICU of Health Sciences University Ümraniye Training and Research Hospital between March 2022 and December 2022, were included in this prospective single-center study.

Eighteen patients without active infection were defined as the control group. The study was approved by the local ethics committee of Health Sciences University Ümraniye Training and Research Hospital (B.10.1.THK.4.34.H.GP.0.01/51) and all procedures complied with the Declaration of Helsinki and its later amendments. Informed consent was obtained from all patients and control subjects or their families.

### 2.2. Data and blood sample collection

The clinical, demographic, and laboratory data of the patients were recorded.

The Pediatric Risk of Mortality (PRISM) score was calculated for patients started on ventilator support for respiratory failure in the first 24 h of treatment while the Organ Failure Index (OFI) and Pediatric Logistic Organ Dysfunction (PELOD) score were calculated at 24 and 72 h of treatment.

The saturation/FiO_2_ (S/F) ratio was calculated for patients who received noninvasive respiratory support, the saturation index [OSI: (FiO_2_ × mean airway pressure × 100/SpO_2_] was calculated for those who received invasive mechanical ventilation support, and the vasoactive inotrope score (VIS) was calculated for those who received inotropic drug support at 24 and 72 h of treatment. The relationship between ARDS development and IL-8 and TM levels was evaluated on the first and third days of respiratory support. Immunosuppressed patients, patients using corticosteroids at a dose of more than 1 mg/kg/day for longer than 1 month, and patients with missing medical records were excluded.

IL-8 and TM kits were obtained from Farmasina Medical and Chemical Products Industries and Foreign Trade Ltd. Co. (Ankara, Türkiye) using an ELX800DA device from Diagnostic Automation Inc. (Woodland Hills, CA, USA) and the KC Junior software program (Agilent Technologies, Santa Clara, CA, USA). Venous blood samples were collected for serum IL-8 and TM levels at 24 and 72 h of respiratory support treatment, simultaneously with samples taken for other tests. After the samples were centrifuged at 4000 rpm for 10 min, the sera were separated into Eppendorf tubes and stored at −80 °C until biochemical analyses. Venous blood samples obtained from the control group were also centrifuged and stored at −80 °C.

### 2.3. Statistical methods

Statistical analyses were performed using IBM SPSS Statistics 22 (IBM Corp., Armonk, NY, USA). The normality of distribution of the study parameters was tested using the Kolmogorov–Smirnov test. Descriptive statistics were calculated, including mean, standard deviation, and frequency values. Nonnormally distributed continuous variables were compared between the study groups using the Kruskal–Wallis test. The Student t-test was used to compare normally distributed parameters between two groups while the Mann–Whitney U test was used to compare nonnormally distributed parameters. Categorical parameters were compared using the Fisher exact chi-square test and continuity (Yates) correction. Multivariate analysis was carried out with logistic regression analysis. When the effects of NIMV/MV, sepsis, ARDS, inotrope requirement, days of PICU stay, first-day IL-8 and TM levels, first- and third-day PRISM scores, first- and third-day PELOD scores, first- and third-day OFI scores, first- and third-day OSI scores, third-day total protein, C-reactive protein (CRP), procalcitonin (PCT), and first- and third-day platelet (PLT) levels on mortality were evaluated by backward stepwise logistic regression analysis, the model was found to be significant (p = 0.001; p < 0.05). The Nagelkerke R^2^ value was 0.755 and the explanatory coefficient of the model (88.2%) was found to be at a good level.

The best cut-off points were determined using receiver operating characteristic (ROC) curve analysis. Pearson correlation analysis was used to test correlations between normally distributed parameters and Spearman rho correlation analysis was used to test correlations between nonnormally distributed parameters. Statistical significance was set at p < 0.05.

## 3. Results

### 3.1. Baseline characteristics of patients

This study was performed with a total of 55 patients, 52.7% (n = 29) of whom were female while 47.3% (n = 26) were male, aged between 1 month and 17 years. The mean age of the patients was 64.16 ± 66.21 months. For respiratory support, 38.2% (n = 21) of the patients received NIMV and 61.8% (n = 34) received IMV. The clinical characteristics of the patients are summarized in Table 1. Clinical signs of sepsis and ARDS were present in 54.5% (n = 30) and 29.1% (n = 16) of the patients, respectively. ARDS was mild in 12.5%, moderate in 43.8%, and severe in 43.8% of patients. The etiologies of patients with ARDS included respiratory failure, sepsis, and bronchopneumonia, whereas etiologies of patients without ARDS included multisystem inflammatory syndrome in children (MIS-C), cardiac diseases, neurological diseases, and sepsis. An underlying disease was found in 81.8% (n = 45) of the patients, 47.3% (n = 26) of all patients were admitted to the PICU for respiratory failure, and 72.7% (n = 40) of patients underwent blood product transfusions. Failure of an organ occurred in 49.1% of patients, failure of two organs in 38.2%, failure of three organs in 10.9%, and failure of 4 organs in 1 patient. Inotropic drug infusion was required in 27.3% of cases. The survival rate of the patients was 76.4%. The patient and control groups were compared with respect to age and sex and no significant difference was found between the two groups.

### 3.2. Changes in parameters between the 24th and 72nd hours

Table 2 presents comparisons of the patients’ biochemical and blood gas parameters and scores on the first and third days. The decreases in CRP, PCT, white blood cell count, alanine aminotransferase, aspartate aminotransferase, urea, creatinine, lactate, and partial carbon dioxide pressure (PaCO_2_) seen on the third day compared to the first day were statistically significant. Significant differences were also found in the PRISM, PELOD, OFI, and OSI scores on the third day compared to the first day.

### 3.3. Serum levels of IL-8 and TM

**Figure 1:**
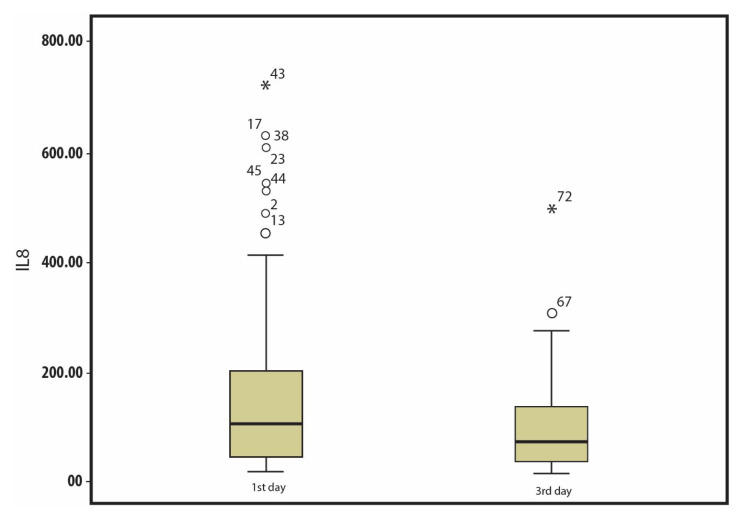
IL-8 levels of patients on the first and third days.

**Figure 2:**
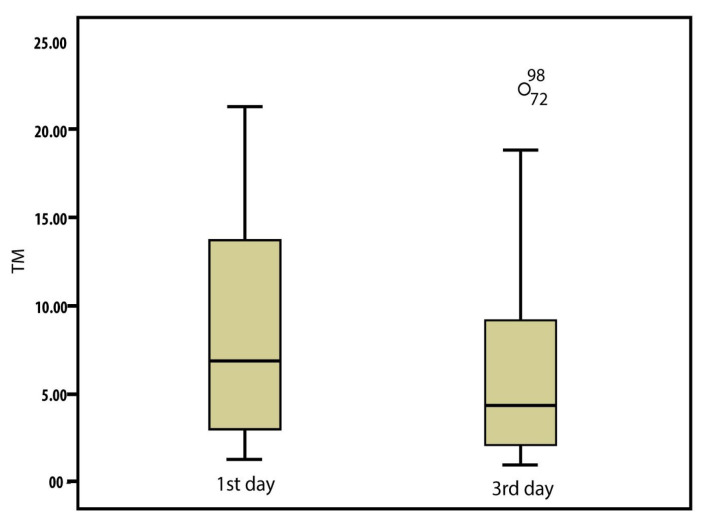
TM levels of patients on the first and third days.

IL-8 and TM levels of the patient group were significantly higher than those of the control group [median IL-8: 102.7 (IQR: 180.42–189.47) vs. 45.4 (55.14–70.49) ng/L, p = 0.011; median TM: 6.9 (6.83–9.18) vs. 3.4 (3.62–5.05) ng/mL, p = 0.021]. As shown in Figures 1 and 2, serum IL-8 and TM levels on the third day were significantly lower than those on the first day. There was no significant difference between the patients who received NIMV and those who received IMV with respect to IL-8 and TM levels on the first and third days. As presented in Table 3, both IL-8 and TM levels of the patients with ARDS were significantly higher than those of patients without ARDS. The median IL-8 levels on the first and third days of patients with ARDS were 176.6 (303.75 **±** 223.08) and 90.2 (118 **±** 76.55) ng/L, respectively, and the IL-8 levels on the first and third days of patients without ARDS were 61.5 (129.82 **±** 149.46) and 43.2 (85.64 **±** 93.62) ng/L, respectively. The median TM levels on the first and third days of patients with ARDS were 12.3 (13.85 **±** 6.81) and 7.2 (9.42 **±** 6.49) ng/mL, respectively, and the TM levels on the first and third days of patients without ARDS were 4.1 (7.26 **±** 5.92) and 3.3 (5.4 **±** 4.85) ng/mL, respectively. There was no significant difference between IL-8 and TM levels according to the presence of sepsis or the ARDS classification. No significant difference was found between IL-8 and TM levels on the first and third days of patients with and without sepsis.

### 3.4. Performance of biomarkers in predicting mortality

Table 4 presents comparisons of the patients’ clinical characteristics and laboratory parameters according to prognosis. There were significant positive relationships of a moderate degree between IL-8 level on the first day and the number of organ failures, PELOD score, and aspartate aminotransferase level. There were moderately strong positive correlations between TM level on the first day and the number of organ failures, aspartate aminotransferase, and activated partial thromboplastin time. There were weak negative correlations between TM level on the first day and the levels of albumin and total protein. Deceased patients had significantly higher TM levels on the first day than survivors, but no significant difference was found for TM levels on the third day.

**Figure 3:**
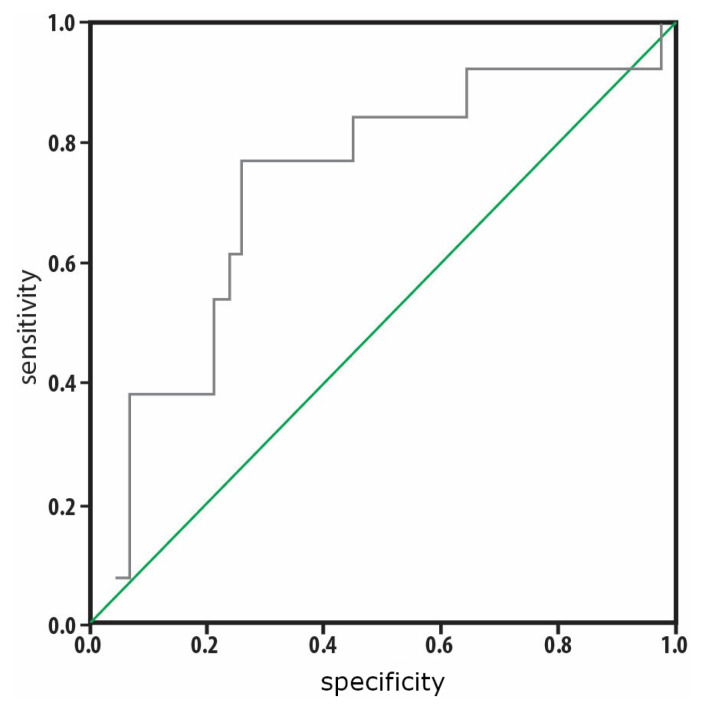
The cut-off point determined for IL-8 level on the first day for mortality prediction was >154.7 ng/L. This level had sensitivity of 76.9% and specificity of 73.8%.

**Figure 4:**
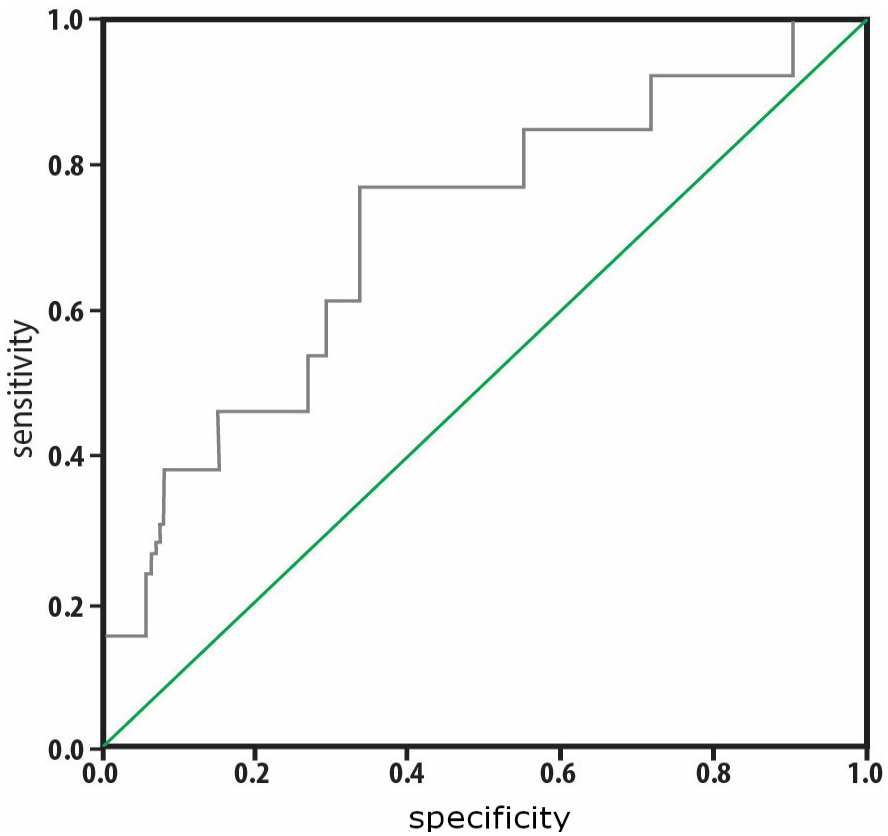
The cut-off level determined for TM on the first day for mortality prediction was >8.4 ng/mL. This level had sensitivity of 76.9% and specificity of 66.7%.

Compared to surviving patients, deceased patients had a higher number of days spent in the PICU and higher NIMV/IMV rates, CRP, PCT, and PRISM, PELOD, OSI, and OFI scores on the third day, although they had lower total protein levels on the third day and lower PLT on the first and third days. The two groups had no significant differences with respect to other parameters. Risk factors affecting mortality are presented in Table 5. Logistic regression analysis was performed to determine the risk factors affecting mortality. The effects of IL-8 level on the first day, PLT on the third day, OFI score on the first day, and OSI score on the first day were statistically significant in the model. Mortality risk was increased 1.017-fold by high IL-8 on the first day, 0.972-fold by low PLT on the third day, 11.418-fold by high OFI score on the first day, and 2.733-fold by high OSI score on the first day. As shown in Figure 3, according to the ROC analysis, the area under the curve (AUC) of the IL-8 level was found to be 0.723 (95% CI: 0.558–0.888). The cut-off point of the IL-8 level for mortality prediction was found to be >154.7 ng/L. This cut-off value had sensitivity of 76.9% and specificity of 73.8%. As shown in Figure 4, according to the ROC analysis, the AUC of the TM level was found to be 0.715 (95% CI**:** 0.542–0.882). The cut-off point of the TM level for mortality prediction was found to be >8.4 ng/mL. This cut-off value had sensitivity of 76.9% and specificity of 66.7%.

## 4. Discussion

TM is an endothelial and pulmonary capillary transmembrane protein with active roles in coagulation and inflammation. Its circulating level is normally very low but increases in inflammatory conditions such as sepsis or ARDS [6]. It is a candidate biological marker for respiratory failure and ARDS. TM plays an important role in the development of the lungs; it increases in response to endothelial injury of different organs, although it is most commonly expressed in the lungs. In a study conducted in adults with acute respiratory distress, increased TM level was shown to correlate with higher mortality [7].

In a study involving 432 pediatric patients treated with invasive mechanical ventilation for acute respiratory failure, Monteiro et al. [8] reported that TM levels ranged between 16.6 and 670.9 ng/mL in the first 5 days of intubation, and an increased TM level was associated with an increased 90-day mortality rate and worse OI. They also reported that both initial TM levels and TM levels during follow-up correlated with extrapulmonary multiorgan failure risk and the severity of hypoxic respiratory failure, and that increased TM level reflected increased dead-space ventilation in patients with ARDS. These findings suggested that vascular injury plays a role in the pathogenesis of acute respiratory failure and it can provide a potential contribution to the determination of treatment targets. Our study also showed that TM levels measured in the first 3 days of intubation were significantly higher in patients with ARDS [12.3 (13.85 **±** 6.81) vs. 7.2 (9.42 **±** 6.49) ng/mL] who underwent invasive mechanical ventilation compared to patients without ARDS [4.1 (7.26 **±** 5.92) vs. 3.3 (5.4 **±** 4.85) ng/mL].

Orwoll et al. [9], in a prospective study involving 243 pediatric patients diagnosed with lung injury, reported that increased soluble TM level was associated with increased mortality and a greater rate of organ dysfunction. In accordance with the literature, our study found a moderately strong (44%), positive, and statistically significant correlation between increased TM level and the number of failed organs. Similarly, TM level measured at 24 h correlated with increased mortality rate (Figure 2).

A study published in 2017, conducted with previously healthy pediatric patients admitted to a PICU for septic shock, reported that TM levels measured on the first and third days were significantly higher in the patient group than the healthy control group. Similarly, the deceased patients had significantly higher TM levels than survivors (9.9 mU/mL vs. 4.4 mU/mL, p = 0.046). There were positive correlations between serum TM levels and PRISM, PELOD, P-MODS, and disseminated intravascular coagulation (DIC) scores on the first day [10]. Similarly, our study found that the patient group had significantly higher TM levels on the first day than the control group [6.9 (6.83–9.18) vs. 3.4 (3.62–5.05) ng/mL, respectively; p = 0.021]. However, no significant correlations were found between TM levels and the PRISM, PELOD, and OFI scores on the first day or sepsis.

Previous animal experiments have shown that recombinant TM has a protective effect in septic mice by suppressing leukocyte adhesion in the microvascular space, reducing thrombus formation, and preventing endothelial injury [11]. Data from another study in mice suggested that TM has protective properties in LPS-mediated ARDS [12].

Randomized controlled studies with adult patients with DIC have compared treatment with recombinant soluble TM and heparin; it has been reported that treatment with TM provided better improvement [13]. In a study of pediatric patients with acute respiratory failure who were intubated and received respiratory support with mechanical ventilation, Carlton et al. reported no significant correlation between functional deterioration and serum IL-8 and TM levels on the first day of intubation, but serum TM levels were higher on the second and third days in patients with deteriorated functional status than those without [14].

IL-8 secretion is induced by TNF and IL-1, which are classical proinflammatory cytokines secreted in the early stages of inflammation. IL-8 is secreted by pulmonary endothelial cells in injuries caused by toxins or infections. It was determined that IL-8 concentrations in bronchoalveolar lavage were 5- to 10-fold higher in intubated patients compared to a control group [15]. Correlations were shown between increased IL-8 levels in both plasma and bronchoalveolar lavage fluid and death and multiorgan failure in adult ARDS patients [16]. It is thought that serially measured IL-8 levels can be used as a marker of treatment efficacy in pediatric patients with sustained inflammation, particularly those with ARDS and respiratory failure. In a multicenter study conducted by Zinter et al. with pediatric ARDS patients, it was determined that IL-6, IL-8, IL-10, and TNF-R2 strongly correlated with mortality, and there were positive correlations between these biomarkers and OI and PRISM scores [17]. Our study found significantly higher IL-8 levels in the patient group than the control group [median IL-8: 102.7 (180.42 **±** 189.47) vs. 45.4 (70.49 **±** 55.14) ng/L, respectively; p = 0.001]. Serum IL-8 level measured on the first day was significantly higher in patients with ARDS than those without [176.6 (303.75 **±** 223.08) vs. 61.5 (129.82 **±** 149.46) ng/L, respectively; p = 0.001].

Flori et al. conducted a prospective study of 480 pediatric patients intubated for respiratory failure. In that study, which was conducted in 22 PICUs, the authors aimed to evaluate the relationship between plasma IL-8 levels measured serially in the early stage and ARDS development and other markers of prognosis in pediatric patients mechanically ventilated for acute respiratory failure. They reported that the highest IL-8 level was determined on the first day of intubation, and IL-8 levels gradually decreased during follow-up. An analysis based on patient subgroups revealed the highest levels in the sepsis group and the lowest levels in the asthma group. Serum IL-6 levels were higher in patients with ARDS than those without. Similarly, it was reported that serum IL-8 levels were 4- to 12-fold higher in deceased patients compared to surviving patients. Serum IL-8 levels were significantly correlated with mortality, duration of mechanical ventilation, and number of days spent in the PICU but not with ARDS development [18]. In our study, patients with respiratory failure received respiratory support by means of NIMV or IMV. No significant difference was found between patients treated with NIMV or IMV regarding IL-8 levels on the first and third days. Unlike other studies, IL-8 levels was found to be significantly higher in patients who developed ARDS. The highest IL-8 level was observed on the first day of intubation (180.4 ng/L); a decrease occurred on the third day (95.05 ng/L). There was no significant difference between patients with or without sepsis. No significant correlation was found between the number of days spent in the PICU and serum IL-8 level.

The limitations of our study include its single-center design and the lack of evaluation of the relationships between viral and bacterial pathogens detected by respiratory tract viral multiplex examination and tracheal aspirate culture proliferations and serum TM and IL-8 levels. Another limitation of our study is that the results cannot be generalized to the immunocompromised patient population as such cases were excluded from the study.

## 5. Conclusion

In this study, higher TM and IL-8 levels in pediatric patients who received invasive or noninvasive respiratory support for respiratory failure were found to correlate with impaired oxygenation, higher mortality, and a higher number of failed organs. Higher TM and IL-8 levels in ARDS might reflect the degree of vascular injury and inflammation. A gradual decline in IL-8 and TM levels during follow-up suggests that these parameters can be used as biomarkers for both determining treatment objectives and predicting prognosis. However, larger studies are needed to use TM and IL-8 as biomarkers.

## Figures and Tables

**Table 1:** Clinical characteristics of the patients.

Study	Min–max	Mean ± SD	Median
PICU (days)	3–123	24.29 ± 28.09	13
NIMV/IMV days (n)	3–123	18.65 ± 25.37	8
VIS	0–240	9.92 ± 34.92	0
		**N**	**%**
Admission diagnosis	Respiratory failure	26	47.3
	Sepsis	21	38.2
	MIS-C	3	5.5
	Status epilepticus	2	3.6
	Postoperative	2	3.6
	Arrhythmia	1	1.8
Underlying disease	Yes	45	81.8
	No	10	18.2
Sepsis	Yes	25	45.5
	No	30	54.5
NIMV/IMV	NIV	21	38.2
	IMV	34	61.8
ARDS	Yes	39	70.9
	No	16	29.1
ARDS severity (n=16)	Mild	2	12.5
	Moderate	7	43.8
	Severe	7	43.8
Blood product	No	15	27.3
	Yes	40	72.7
Number of failed organs	1	27	49.1
	2	21	38.2
	3	6	10.9
	4	1	1.8
Inotrope	No	40	72.7
	Yes	15	27.3
Mortality	Survived	42	76.4
	Deceased	13	23.6

PICU (days): Number of days spent at pediatric intensive care unit, NIMV/IMV days (n): number of days on noninvasive mechanical ventilation/mechanical ventilation, VIS: vasoactive inotrope score.

**Table 2:** Comparison of the patients’ biochemical and blood gas parameters and scores on the first and third days.

	1st day	3rd day	
	Mean ± SD (median)	Mean ± SD (median)	p
IL-8 (ng/L)	180.42 ± 189.47 (102.7)	95.05 ± 89.53 (70.5)	10.001^*^
TM (ng/mL)	9.18 ± 6.83 (6.9)	6.57 ± 5.62 (4.4)	10.001^*^
ALT (U/L)	128.51 ± 496.87 (28)	61.75 ± 126.54 (23)	10.046^*^
AST (U/L)	239.47 ± 836.26 (48)	75.31 ± 141.27 (28)	10.001^*^
BUN (mg/dL)	28.53 ± 28.29 (17.9)	20.57 ± 20.96 (13.6)	10.002^*^
Creatinine (mg/dL)	0.42 ± 0.37 (0.3)	0.35 ± 0.3 (0.2)	10.009^*^
Calcium (mg/dL)	8.76 ± 0.71 (8.8)	9.01 ± 0.67 (9)	10.003^*^
CRP (mg/L)	64.18 ± 79.64 (33)	39.14 ± 53.63 (21)	10.001^*^
PCT (ng/mL)	11.26 ± 24.97 (0.8)	3.25 ± 6.82 (0.5)	10.001^*^
WBC (10^3^/μL)	11.82 ± 8.32 (11.5)	9.56 ± 6.41 (8.8)	10.003^*^
pH	7.29 ± 0.17 (7.3)	7.39 ± 0.09 (7.4)	20.001^*^
PCO_2_ (mm/Hg)	49.92 ± 20.88 (46.7)	42 ± 11.25 (40.8)	20.009^*^
HCO_3_ (mmol/L)	21.63 ± 5.88 (22.4)	24.7 ± 4.45 (24)	20.001^*^
Lactate (mmol/L)	3.22 ± 3.6 (1.8)	2.21 ± 3.73 (1.3)	10.023^*^
PRISM	9.42 ± 5.14 (9)	6.44 ± 5.12 (7)	0.001^*^
PELOD	13.67 ± 7.81 (11)	8.89 ± 6.34 (10)	0.001^*^
OFI	1.6 ± 1.12 (2)	1.22 ± 1.17 (1)	0.001^*^
OSI	9.2 ± 3.81 (9.1)	7.79 ± 4.12 (6.8)	0.024^*^
S/F	197.28 ± 47.82 (169)	271.24 ± 30.60 (270)	0.001^*^

1 Wilcoxon sign test

2 Paired samples t test

^*^ *p < 0.05*

ALT: alanine aminotransferase, AST: aspartate amino transaminase, BUN: blood urea nitrogen, CRP: C reactive protein, PCT: procalcitonin, WBC: white blood cell count, PaCO_2_: partial arterial carbon dioxide pressure, HCO_3:_ bicarbonate PRISM: Pediatric Mortality Risk Scoring, PELOD: Pediatric Logistic Organ Dysfunction Score, OFI: Organ Failure Index, OSI: Oxygen Saturation Index, S/F: saturation/FiO_2_.

**Table 3:** Comparisons of the IL-8 and TM levels of the patients.

		ARDS absent	ARDS present	
		Mean ± SD (median)	Mean ± SD (median)	p
IL-8 (ng/L)	1st day	129.82 ± 149.46 (61.5)	303.75 ± 223.08 (176.6)	0.001^*^
	3rd day	85.64 ± 93.62 (43.2)	118 ± 76.55 (90.2)	0.020^*^
TM (ng/mL)	1st day	7.26 ± 5.92 (4.1)	13.85 ± 6.81 (12.3)	0.001^*^
	3rd day	5.4 ± 4.85 (3.3)	9.42 ± 6.49 (7.2)	0.006^*^

**Table 4:** Comparisons of the patients’ clinical characteristics and laboratory parameters according to prognosis.

		Survivors	Deceased	
		Mean ± SD (median)	Mean ± SD (median)	p
Age (months)		68.9 ± 69.03 (37)	48.85 ± 55.77 (16)	10.545
Number of days on NIMV/IMV		16.12 ± 25.86 (6.5)	26.85 ± 22.75 (20)	10.002^*^
Number of failed organs		1.55 ± 0.71 (1)	2 ± 0.82 (2)	10.055
IL-8 (ng/L)	1st day	150.53 ± 174.19 (67.9)	276.98 ±211.3 (173.3)	10.016^*^
	3rd day	89.63 ± 93.69 (51.2)	112.56 ± 75.13 (83.1)	10.191
TM (ng/mL)	1st day	7.93 ± 6.3 (5.4)	13.2 ± 7.17 (11.8)	10.020^*^
	3rd day	6.09 ± 5.67 (3.7)	8.12 ± 5.4 (7.3)	10.143
PRISM score	1st day	8.62 ± 5.22 (8)	12 ± 4.06 (12)	10.013^*^
	3rd day	5.26 ± 4.54 (2.5)	10.23 ± 5.21 (10)	10.001^*^
PELOD score	1st day	12.5 ± 7.77 (11)	17.46 ± 6.92 (20)	10.008^*^
	3rd day	7.4 ± 6.13 (10)	13.69 ± 4.46 (12)	10.001^*^
OFI score	1st day	1.36 ± 1.03 (1)	2.38 ± 1.04 (2)	10.002^*^
	3rd day	0.93 ± 1 (1)	2.15 ± 1.21 (2)	10.001^*^
OSI	1st day	7.7 ± 3 (8)	11.62 ± 3.82 (12)	10.002^*^
	3rd day	6.18 ± 3.35 (6)	10.39 ± 4.04 (10.2)	10.003^*^
Total protein (g/L)	1st day	54.22 ± 11.62 (54)	50.54 ± 8.68 (51)	20.298
	3rd day	55.42 ± 8.81 (56)	47.8 ± 6.27 (48)	20.006^*^
C Reactive protein (mg/L)	1st day	64.63 ± 83.14 (30.3)	62.75 ± 70.16 (44)	10.866
	3rd day	28.86 ± 35.29 (15.5)	72.36 ± 84.39 (36.8)	10.025^*^
Procalcitonin (ng/mL)	1st day	7.47 ± 19.69 (0.7)	23.48 ± 35.61 (2.2)	10.061
	3rd day	2.2 ± 4.87 (0.3)	6.66 ± 10.57 (1.3)	10.011^*^
PLT (10^3^/μL)	1st day	250.26 ± 138.96 (243)	148 ± 154.93 (82)	20.028^*^
	3rd day	266.81 ± 160.51 (242)	106.77 ± 95.75 (80)	20.001^*^

1 Mann–Whitney U Test

2 Student’s t test

^*^ *p < 0.05*

**Table 5:** Evaluation of risk factors affecting mortality.

		95% confidence interval		
	OR	Lower limit	Upper limit	p
IL-8 (ng/L) (1st day)	1.017	0.999	1.034	0.047^*^
PCT (ng/mL) (3rd day)	0.639	0.366	1.116	0.115
PLT (10^3^/μL) (3rd day)	0.972	0.946	0.999	0.044^*^
PELOD (1st day)	0.657	0.417	1.035	0.070
OFI (1st day)	11.418	0.624	209.042	0.021^*^
OSI (1st day)	2.733	0.887	8.418	0.040^*^

PELOD: Pediatric Logistic Organ Dysfunction Score, OFI: Organ Failure Index, OSI: Oxygen Saturation Index, IL-8: Interleukin-8. PCT: procalcitonin, PLT: platelet.
